# Optimization of Heterotrophic Culture Conditions for the Algae *Graesiella emersonii* WBG-1 to Produce Proteins

**DOI:** 10.3390/plants12122255

**Published:** 2023-06-09

**Authors:** Kaixuan Wang, Zhongjie Wang, Yi Ding, Youzhi Yu, Yali Wang, Yahong Geng, Yeguang Li, Xiaobin Wen

**Affiliations:** 1 CAS Key Laboratory of Plant Germplasm Enhancement and Specialty Agriculture, Wuhan Botanical Garden, Chinese Academy of Sciences, Wuhan 430074, China; wangkaixuan19@mails.ucas.ac.cn (K.W.); jiezhongwang1@163.com (Z.W.); dingyi@wbgcas.cn (Y.D.); yuyouzhi15@mails.ucas.ac.cn (Y.Y.); wangyali20@mails.ucas.ac.cn (Y.W.); yahong@wbgcas.cn (Y.G.); 2 Center of Economic Botany, Core Botanical Gardens, Chinese Academy of Sciences, Wuhan 430074, China; 3 University of Chinese Academy of Sciences, Beijing 100049, China

**Keywords:** *Graesiella emersonii*, heterotrophic, condition optimization, protein production

## Abstract

The aim of this study was to improve the protein content and yield of heterotrophic microalgal cultivation and establish a simple, economical, and efficient method for microalgal protein production using the novel green alga, *Graesiella emersonii* WBG-1, which has not been previously reported for heterotrophic cultivation. Through batch heterotrophic cultivation of this alga, we observed that glucose was the optimal carbon source, while it could not use sucrose as a carbon source. Biomass production and protein content were significantly reduced when sodium acetate was used as the carbon source. Compared with nitrate, protein content increased by 93% when urea was used as the nitrogen source. Cultivation temperature had a significant impact on biomass production and protein content. The optimal conditions were glucose as the carbon source at an initial concentration of 10 g/L, urea as the nitrogen source at an initial concentration of 1.62 g/L, and a culture temperature of 35 °C. On the second day of batch cultivation, the highest protein content (66.14%) was achieved, which was significantly higher than that reported in heterotrophic cultures of *Chlorella* and much higher than that reported for specially established technologies aimed at increasing the protein content, such as two-stage heterotrophic, heterotrophy–dilution–photoinduction, and mixotrophic processes. These results demonstrate the great potential of the heterotrophic cultivation of *G. emersonii* WBG-1 for protein production.

## 1. Introduction

The continuous growth of the global human population, and the consequent increase in demand for more and better-quality food, further strain the world’s natural resources [1]. Protein is an essential nutrient in the human diet, with an annual global demand of approximately 202 million tons per year, while the current global protein production is approximately 100 million tons per year [2]. This significant gap between protein production capacity and demand inevitably raises concerns regarding food security [2]. Significant efforts have been made to explore new sources to meet the increasing demand for protein. Microalgae can synthesize all necessary amino acids, and the average protein content of most microalgae is higher than that of traditional plant protein sources [3] Therefore, microalgae are a relatively new and promising source of protein. Furthermore, due to the technological feasibility of protein production by microalgae and their application in the food and feed industries, this new protein source can contribute to addressing the global protein supply shortage [4,5].

As photoautotrophs, microalgae are called “multifunctional cell factories” that produce potentially valuable protein molecules for food, medicine, and feed [6,7]. Currently, photoautotrophic culture in open raceway ponds and closed tubular bioreactors are the traditional and common methods for *Chlorella* and *Spirulina* cultivation. Algal biomass with a 50–70% protein content on a dry weight basis is produced and used for human consumption and animal feed [8,9]. However, under photosynthetic autotrophic conditions, low microalgal biomass leads to low protein productivity, and the risk of microbial contamination reduces production efficiency [10]. To address these issues, researchers have developed heterotrophic cultures of microalgae in which light is not involved and, instead, organic carbon sources are used [11,12]. In the heterotrophic culture mode, microalgae can use glucose efficiently to achieve high growth rates and accumulate large amounts of biomass [13,14]. Therefore, the heterotrophic culture mode enables microalgae to produce biomass with a high protein content, and is a promising alternative to the conventional autotrophic culture of microalgae.

Although current methods of the heterotrophic culture of microalgae already achieve higher biomass densities than photoautotrophic culture [15,16], they face a serious challenge: the protein content (generally below 40%) is significantly lower than that produced through photoautotrophic culture [17]. Researchers have explored various methods to enhance protein synthesis in microalgae and increase their protein content in heterotrophic cultures. Thus, for example, Ogbonna developed a sequential heterotrophic–autotrophic culture method to achieve high biomass and high protein content (60.1%) by supplying light and passing CO_2_ into the culture at the end of the heterotrophic culture of *Chlorella vulgaris* [18]; Mahboob et al. [19] reported that a mixotrophic culture mode was applied to cultivate *C. vulgaris* in a 10 L fermenter and achieved a protein content of 60.0% on a dry weight basis. Furthermore, Fan et al. [20] established a sequential heterotrophic–dilution–photoinduction cultivation scheme. In this case, after reaching a high cell density in the heterotrophic culture of *C. vulgaris*, the algal suspension was diluted to a suitable concentration and transferred to light conditions for photoinduction to increase intracellular protein in *C. vulgaris* by 50.87%. The methods for increasing the density of algal cells by heterotrophic culture and then transferring algal cells into autotrophic conditions or mixotrophic conditions can improve the protein content of *Chlorella*; however, these methods are difficult to apply in practice, mainly due to the following two aspects: (1) Heterotrophic culture followed by autotrophic or mixotrophic culture is easy to implement in the laboratory, but distributing enough light energy to each algal cell is a great challenge when culture is scaled up, as algal cells cannot obtain sufficient light to conduct autotrophic growth effectively; because the population density of algal cells in heterotrophic/mixotrophic culture is high, the intensity of supplemental light declines rapidly; (2) in the heterotrophic–dilution–photoinduction process, the photoautotrophic culture facilities are an indispensable part of the culture system; additionally, culture time is prolonged, which makes the technology more complex and increases the risk of contamination of the culture. Recently, researchers explored a method to promote algal protein synthesis based on the theory of “compensatory growth”. Xie et al. [21] incubated *C. vulgaris* in a heterotrophic mode in which the alga grew first under conditions of unlimited nitrogen supply to accumulate biomass, followed by a period under nitrogen-deficient conditions; then, a high amount of nitrogen was supplemented to induce the “compensatory uptake” of N, by which the synthesis of protein was promoted. However, the protein content of *C. vulgaris* reached only 44.3% using this method. Previous studies have shown that protein accumulation depends on the selected microalgal species [22]. As all methods reported for increasing protein content have problems, they cannot realize economic and efficient algal protein production. Therefore, a better practical strategy to achieve this goal might include using algal strains with high protein content in heterotrophic conditions and optimizing culture conditions.

*Graesiella emersonii* is a unicellular green alga. In previous studies, this alga has mainly been investigated in terms of its lipid production characteristics under photosynthetic autotrophic culture conditions [23,24]. The protein content of *G. emersonii* was 37.07% in a photosynthetic autotrophic culture [25]. So far, *G. emersonii* has not been reported as a protein producer under heterotrophic culture. In this study, the algal strain *G. emersonii* WBG-1, which shows a rapid growth rate and high protein content, was used for heterotrophic culture. This algae was previously collected and selected in our laboratory and can grow autotrophically, heterotrophically, and mixotrophically. In addition, the cells of *G. emersonii* WBG-1 are much larger than those of *Chlorella*, which makes them much easier to harvest via natural sedimentation [26]. *G. emersonii* WBG-1 has been successfully cultivated in 1000 m^2^ open-raceway ponds in a mixotrophic mode with acetate as the carbon source and is considered a promising algal strain for mass protein production [27]. In the pre-experiments, we found that the protein content of *G. emersonii* WBG-1 was higher in heterotrophic than in photoautotrophic cultures. This study aimed to establish a simple, economical, and efficient heterotrophic technology for microalgae protein production by optimizing culture conditions, specifically carbon and nitrogen sources and growth temperature, for the heterotrophic cultivation of *G. emersonii* WBG-1.

## 2. Results and Discussion

### 2.1. Effects of Carbon Source and Initial Concentration on Growth and Protein Yield of G. emersonii WBG-1

Heterotrophic microalgae use organic carbon sources for growth and reproduction. Carbon is the main component of microalgae, accounting for 17.5–65% of their total dry weight, depending on the species and culture conditions [28]. The carbon content of *G. emersonii* WBG-1 was about 45% of dry weight. To obtain the most suitable carbon source for the heterotrophic culture of *G. emersonii* WBG-1 for protein production, the effects of glucose, sodium acetate, and sucrose on the growth and protein content of heterotrophic *G. emersonii* WBG-1 were comprehensively evaluated. The results are summarized in Table 1.

Glucose facilitated cell growth and protein production of the alga, with a maximum biomass concentration of 15.36 g/L and protein content and yield of 34.19% and 5.25 g/L, respectively. In contrast, although 30 g/L sodium acetate supported algal growth, biomass production, and protein yield, they were significantly lower than those obtained with 30 g/L glucose. In addition, in our preliminary experiments, *G. emersonii* WBG-1 was cultured with lower concentrations of sodium acetate (5 g/L and 10 g/L), the results showed significant lower growth rates and biomass concentrations compared to the cultures with the same concentration of glucose. This result is consistent with previous studies. For example, Heredia-Arroyo et al. [29] reported that *Chlorella protothecoides* showed significantly higher biomass accumulation in heterotrophic cultivation when glucose was used as the carbon source compared to sodium acetate. Similarly, Kim et al. [30] found that *Chlorella* sp. HS2 accumulated more biomass when glucose was used as the carbon source compared to sodium acetate. Furthermore, *G. emersonii* WBG-1 did not grow when sucrose was used as the carbon source. This is consistent with previous findings that under heterotrophic cultivation, most of the investigated algal species were not able to grow on sucrose owing to a lack of extracellular sucrose hydrolysis activity and sucrose transport activity [31]. Glucose contains more energy than other organic substrates and is the best carbon source for most microalgal heterotrophic cultures [32,33]. This finding is consistent with our results.

**Figure 1:**
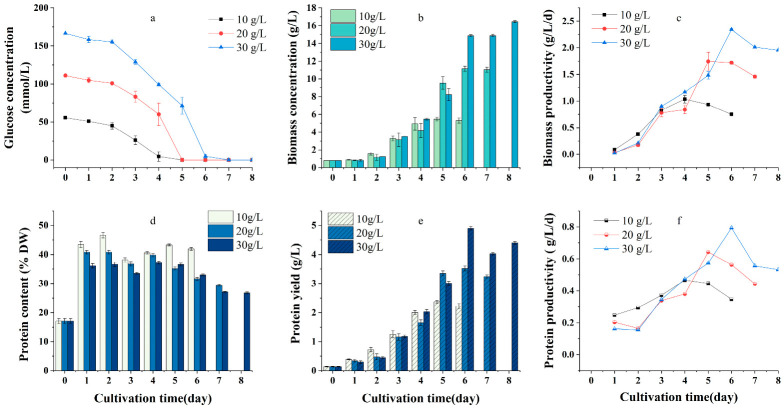
Effect of initial concentrations of glucose on biomass and protein production of *Graesiella emersonii* WBG-1. Variation of glucose concentration in the culture medium (**a**), time evolutions of biomass concentration (**b**), and biomass productivity (**c**). time evolutions of protein content (**d**), protein yield (**e**), and protein productivity (**f**). Error bars represent standard deviation (n = 3).

The effects of initial glucose concentrations of 10 g/L, 20 g/L, and 30 g/L on cell growth, protein content, and yield were evaluated, with the corresponding initial urea concentrations of 1.56 g/L, 3.12 g/L and 4.68 g/L, respectively. The same C/N ratio (atomic ratio of initial carbon concentration to initial nitrogen concentration) was maintained. The results are shown in Figure 1.

The results showed the same trend in glucose consumption at the three initial glucose concentrations tested during culture. During the first two days of culture, carbon consumption was slow, but then the algal cells began to grow rapidly, absorbing carbon to support biomass accumulation. The carbon source was depleted on days four, five, and seven after culture initiation, with initial carbon concentrations of 10, 20, and 30 g/L, respectively (Figure 1a). The efficiency of glucose conversion to biomass was approximately 55%, and no significant differences (*p* > 0.05) were observed among the three initial carbon concentrations tested. The maximum biomass concentration and biomass productivity gradually increased with increasing initial glucose concentrations, and the difference among glucose treatments was significant (*p* < 0.01). At an initial glucose concentration of 30 g/L, biomass and biomass productivity peaked at 16.43 g/L and 2.34 g/L/d, respectively (Figure 1b).

Intracellular protein content exhibited a consistent trend among the different initial glucose concentrations during cultivation (Figure 1c). Thus, when glucose was depleted, the protein content of the culture at an initial glucose concentration of 10 g/L was significantly higher than that observed at the other two initial glucose concentrations (*p* < 0.01). The intracellular protein content was 43.25% on day five after treatment initiation. The changes in protein yield and productivity are shown in Figure 1e,f; they reached a maximum value when the carbon source was depleted. The protein productivity in the culture under an initial glucose concentration of 10 g/L was higher than that obtained under other initial glucose concentrations before carbon was depleted. Safi C reported that excess carbon led to the inhibition of *Chlorella* protein synthesis [34], which is consistent with the results of this study. The biomass productivity, protein content, and productivity decreased with increasing glucose concentration over the first two days of culture (Figure 1c,d,f), probably because of the increased inhibitory effect of high glucose concentration on cell growth.

Previous studies have shown that high glucose concentrations increased the biomass concentration of *Chlorella* significantly, but too much glucose may generate osmotic pressure which had an inhibitory effect on the growth of *Chlorella* cells [35,36]. In this study, no significant difference was observed in the conversion rate of glucose to biomass at different initial glucose concentrations; however, the intracellular protein content decreased significantly with increasing initial glucose concentration. Considering that a high initial carbon concentration is not necessary for algal heterotrophic production technology, in which the carbon is usually fed continuously to ensure the supplementary material, it is important to choose the initial carbon concentration that helps reach a higher protein content and yield before carbon is depleted. Considering biomass productivity and protein content, 10 g/L of glucose was selected as the optimal initial carbon concentration for all subsequent experiments.

### 2.2. Effects of Nitrogen Sources and Initial Nitrogen Concentration on the Growth, Protein Content, and Yield of G. emersonii WBG-1

**Figure 2:**
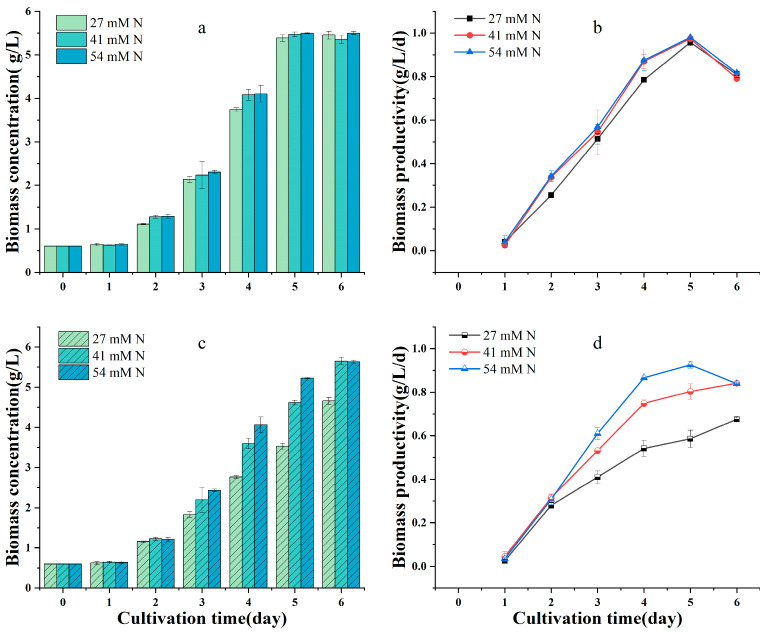
Effect of nitrogen sources and initial concentrations on the growth of *Graesiella emersonii* WBG-1. (**a**,**b**), Urea as a nitrogen source, (**c**,**d**), nitrate as a nitrogen source. Error bars represent standard deviation (n = 3).

Nitrogen is an essential macronutrient for microalgae, and an appropriate N supply is important for increasing protein content and yield. Microalgae can utilize various nitrogen sources, such as urea and sodium nitrate, among others; however, the biomass concentration and protein content can be significantly affected by the type and concentration of nitrogen source used [37,38]. To screen the best nitrogen source for protein production by the heterotrophic culture of *G. emersonii* WBG-1, the effects of two nitrogen sources (urea and mixed nitrate) and their initial concentrations on the growth, protein content, and yield were investigated under an initial glucose concentration of 10 g/L. The results are shown in Figure 2.

*G. emersonii* WBG-1 was able to grow at all of the different concentrations of urea tested, and the maximum biomass reached was approximately 5.5 g/L, regardless of urea concentration (Figure 2a), with no significant difference in biomass productivity (*p* > 0.05) (Figure 2b). In contrast, the initial nitrate concentration significantly affected algal growth. Biomass concentration (Figure 2c) and productivity (Figure 2d) increased with increasing initial N concentration. This finding is in line with previous research, which showed that, as the nitrate concentration increased, the growth rate of *Chlorella sorokiniana* in heterotrophic cultivation also increased [39]. Thus, in the culture under an initial nitrate concentration of 54 mM, the biomass concentration and productivity were significantly higher on days 3–5 than those under initial nitrate concentrations of 27 and 41 mM.

**Figure 3:**
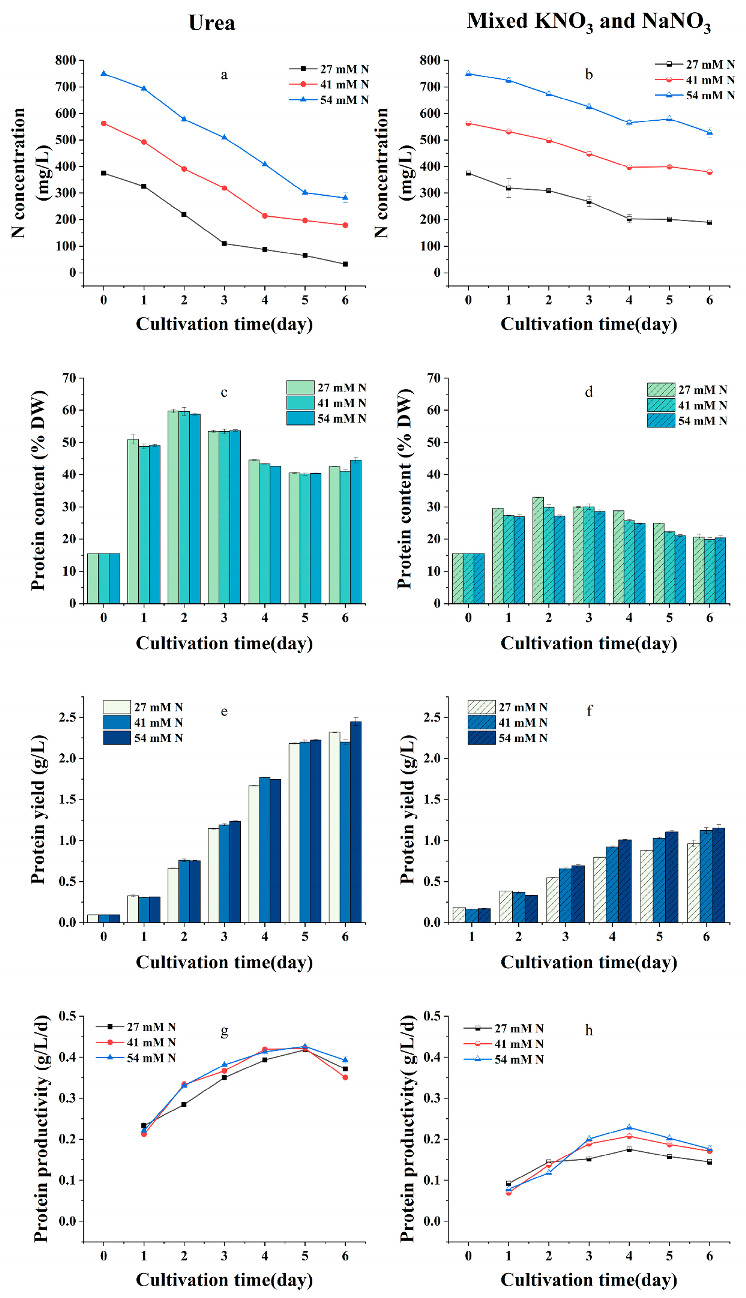
Effect of nitrogen sources and initial concentrations on protein content and yield of *Graesiella emersonii* WBG-1. (**a**,**c**,**e**,**g**) urea as a nitrogen source, (**b**,**d**,**f**,**h**) nitrate as a nitrogen source. Error bars represent standard deviation (n = 3).

Figure 3 demonstrates the effects of nitrogen sources and initial concentrations on the protein content and yield. The protein content, yield, and productivity with different initial nitrogen concentrations showed the same trend during the incubation (Figure 3c,e,g). Maximum protein content (44.53%), yield (2.45 g/L), and productivity (0.39 g/L/d) were obtained at an initial N concentration of 54 mM at the end of the culture period (day 6), the maximum protein content, yield, and productivity were significantly higher than the corresponding values under the initial nitrogen concentrations of 27 and 41 mM (*p* < 0.05). In cultures with urea as the nitrogen source at initial N concentrations of 27, 41, and 54 mM (corresponding to urea concentrations of 13.5, 20.5, and 27 mM, respectively), the algae assimilated 24.5, 27.4, and 33.4 mM nitrogen, respectively (Figure 3a), and nitrogen uptake increased with increasing initial nitrogen concentration (*p* < 0.05).

In cultures with initial nitrate concentrations of 27, 41, and 54 mM, the algae assimilated 13.2, 13.1, and 15.9 mM nitrogen, respectively (Figure 3b). The protein content at different nitrate concentrations initially increased and then decreased during incubation (Figure 3d). The biomass increased significantly with increasing nitrate concentrations (*p* < 0.05), whereas the changes in protein content showed the opposite trend. The effect of nitrate concentration on biomass was stronger than that on protein content; protein yield (Figure 3f) and productivity (Figure 3h) significantly increased (*p* < 0.05). A maximum protein yield of 1.15 g/L was achieved on day four of the culture at an initial nitrate concentration of 54 mM.

Compared with mixed nitrate as a nitrogen source, urea significantly enhanced nitrogen uptake, protein content, protein yield, and productivity (*p* < 0.01), and the protein content increased by 93%, indicating that urea is a more favorable nitrogen source for mass protein production by *G. emersonii* WBG-1. This result is similar to that reported by Lai, in which urea was used as a nitrogen source for the heterotrophic culture of *Chlorella sorokiniana* and the protein content increased from 48.94 to 58.8% compared to when nitrate was used as the nitrogen source [40]. Additionally, it has been reported that microalgal protein synthesis is enhanced by sufficient nitrogen supply [21,40], and this study confirmed that the higher protein yield of *G. emersonii* WBG-1 was achieved at the higher nitrogen concentration (Figure 3e,f). Considering both its effects on biomass production and protein yield, urea was selected as the most favorable nitrogen source, with an initial N concentration of 54 mM (urea concentration of 27 mM).

When nitrate is used as a nitrogen source, algae absorb and transport nitrate into cells by active absorption. Once within the cell, nitrate is enzymatically reduced to nitrite (NO_2_^−^) by nitrate reductase and then to ammonium (NH4^+^) by nitrite reductase to participate in amino acid synthesis. The uptake and reduction of nitrate are energy-consuming processes, especially the reduction process from NO_3_^−^ to NH_4_^+^, which requires large amounts of energy. In contrast, urea is an organic nitrogen that does not require an energy-consuming reduction reaction. Therefore, compared to nitrate, urea is an energy-saving nitrogen source. This may be one of the reasons why organic nitrogen is more beneficial to microalgae for protein production. Notably, the protein content of *G. emersonii* WBG-1 cultured with urea as a nitrogen source was almost double compared to that observed when nitrate was used as a nitrogen source, and the increase (93%) was much greater than that (20%) reported for *Chlorella* [40], indicating that *G. emersonii* WBG-1 is extremely sensitive to nitrogen. Therefore, *G. emersonii* WBG-1 may be an ideal experimental material for studying the effects of organic and inorganic nitrogen on protein content in microalgae.

### 2.3. Effect of Temperature on Heterotrophic Growth, Protein Content, and Yield of G. emersonii WBG-1

Temperature is the most important environmental factor affecting the heterotrophic growth of microalgae. To select the optimal temperature for protein production in heterotrophic cultivation of *G. emersonii* WBG-1, the effect of temperature (25, 28, 31, 33, 35, and 37 °C) on the growth, protein content, and yield of the algae was investigated using glucose as the carbon source at an initial concentration of 10 g/L and urea as the nitrogen source at an initial concentration of 1.624 g/L.

**Figure 4:**
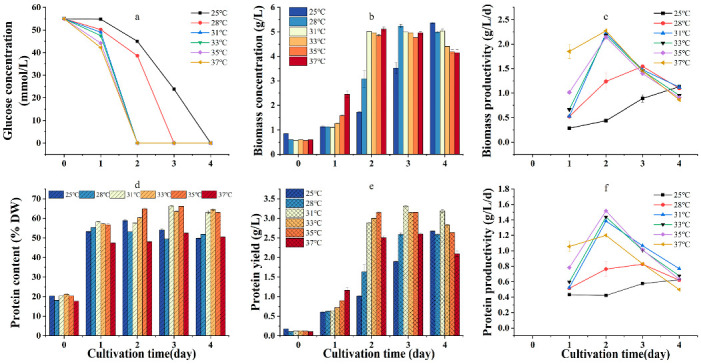
Effects of temperature on the growth and protein production of *Graesiella emersonii* WBG-1. Variation of glucose concentration in the culture medium (**a**), time evolutions of biomass concentration (**b**), and biomass productivity (**c**). time evolutions of protein content (**d**), protein yield (**e**), and protein productivity (**f**). Error bars represent standard deviation (n = 3).

Under experimental conditions, glucose consumption increased with temperature. In this study, at 31 °C, glucose was completely depleted after 48 h of incubation. At 28 and 25 °C, glucose was depleted at 72 h and 96 h, respectively (Figure 4a). During the first two days of culture, algal biomass increased more rapidly as temperature increased, and maximum biomass (approximately 5 g/L) was reached at 48 h after culture initiation at 31 °C and above (Figure 4b). On day four, biomass decreased to a certain extent at all temperatures, except at 25 °C (the lowest temperature tested), probably due to the active respiration of cells as they continuously consume biomass after depletion of organic carbon in the culture medium, which is a common phenomenon in microalgae heterotrophic culture [39]. Figure 4c shows the effect of temperature on biomass productivity. When the temperature was 31 °C or above, biomass productivity peaked at approximately 2.31 g/L/d on day two, with no significant difference among temperature treatments (*p* > 0.05). At 28 and 25 °C, biomass productivity peaked on days three and four, respectively, but was significantly lower than that at 31 °C or above (*p* < 0.05).

The effect of temperature on protein content is shown in Figure 4d. The protein content of *G. emersonii* WBG-1 generally increased with temperature up to 35 °C, but decreased significantly at 37 °C. Protein content and yield at 31–35 °C were significantly higher (*p* < 0.05) than those at 25, 28, and 37 °C. Figure 4f shows the effect of temperature on protein productivity. When the temperature was not below 31 °C, all cultures reached their maximum protein productivity on the second day, and the protein productivity at 35 °C (1.52 g/L/d) was significantly higher than that for the other five temperature treatments (*p* < 0.05). At 28 and 25 °C, the maximal protein productivities were reached on days 3 and 4, respectively. However, they were significantly lower than those at temperatures above 31 °C but below 37 °C (*p* < 0.05).

In algal cells, enzyme activities related to growth, synthesis, and metabolism show optimal temperatures and are negatively impacted by higher or lower temperatures, thus affecting the efficiency of biochemical processes. Previous studies have shown that the optimum temperature for the heterotrophic growth of green algae varies depending on the algal species [41,42]. Thus, for example, Mahboob et al. [19] cultured *C. vulgaris* at temperatures ranging from 25 to 40 °C and obtained a maximum protein yield at 35 °C. In this study, the protein content of *G. emersonii* WBG-1 increased with increasing culture temperature up to 35 °C. However, protein content decreased significantly when the incubation temperature reached 37 °C. Protein productivity reflects the combined effects of temperature on the biomass and protein content of *G. emersonii* WBG-1. Figure 4f shows that maximum protein productivity was achieved at 35 °C. Therefore, the optimum temperature for mass protein production in the heterotrophic culture of *G. emersonii* WBG-1 was 35 °C.

Under optimal conditions in heterotrophic cultures, the protein content of *G. emersonii* WBG-1 reached 66.1%, which was significantly higher than that reported for *Chlorella;* however, it was much higher than that reported in specially established technologies aimed at increasing protein content, such as two-stage heterotrophic [21,37], sequential heterotrophic/autotrophic [18], heterotrophy–dilution–photoinduction [20], and mixotrophic processes [19]. These data are summarized in Table 2. The results indicate that *G. emersonii* WBG-1 is a promising strain suitable for mass protein production in heterotrophic cultures. The protein content was only 34.19% under non-optimized conditions (initial glucose concentration of 30 g/L, initial urea concentration of 6 g/L, temperature of 28 °C); however, it was almost doubled after condition optimization. This study demonstrates the importance of optimizing culture conditions for microalgae to realize their potential for microalgal mass protein production.

## 3. Materials and Methods

### 3.1. Microalgal Strain

The high-protein-yield strain *G. emersonii* WBG-1, a green alga with large globose cells capable of rapid heterotrophic growth, was provided by the Algae Culture Collection of the Wuhan Botanical Garden of the Chinese Academy of Sciences. This strain was originally isolated from Chenghai Lake in Yunnan Province, China. 

### 3.2. Algae Seed Culture Conditions

The heterotrophic algal seed was maintained in a modified Endo growth medium [45], containing 30 g/L glucose, 3 g/L KNO_3_, 1.2 g/L KH_2_PO_4_, 1.2 g/L MgSO_4_·7H2O, 0.2 g/L trisodium citrate, 0.016 g/L FeSO_4_·7H_2_O, 2.1 mg/L EDTA-Na_2_, 0.105 g/L CaCl_2_·2H_2_O, 2.86 mg/L H_3_BO_3_, 0.222 mg/L ZnSO_4_·7H_2_O, 1.81 mg/L MnCl_2_·4H_2_O, 0.021 mg/L Na_2_MoO_4_·2H_2_O, and 0.07 mg/L CuSO_4_·5H_2_O. In addition, the initial pH of the modified Endo medium was adjusted to 6.0 with 1 M NaOH solution.

To prepare the inoculants for heterotrophic culture, a single colony of *G. emersonii* WBG-1 pure culture was inoculated into a 200 mL tissue culture bottle containing 50 mL of modified Endo medium and grown at 28 °C in an incubator (ZQLY-180E, Zhichu, Shanghai, China) at 180 r/min. After 5–6 d of growth, the concentrated algal suspension was inoculated into 500 mL glass flasks containing 200 mL modified Endo medium with an inoculum volume of 1% (*v*/*v*), reaching an initial biomass concentration of 0.5 g/L. The seed culture was grown under the above culture conditions for 6 d, and then used as inoculum for fermentation.

### 3.3. Optimization of the Carbon Source

Optimization of the carbon source (including carbon type and initial concentration) was conducted in 250 mL glass flasks with a working volume of 100 mL by inoculating 10% (*v*/*v*) of the exponentially growing algal suspension, reaching an initial biomass concentration of 0.5 g/L. Different carbon skeletons (glucose, sucrose, and sodium acetate) were tested to select the best carbon source by analyzing their effect on *G. emersonii* WBG-1 growth and protein content, and then different initial concentrations of the selected best carbon source (10, 20, and 30 g/L) were applied to determine the optimal initial concentration by analyzing their effect on *G. emersonii* WBG-1 growth and protein content. The same C/N ratio (atomic ratio of initial carbon concentration to initial nitrogen concentration) was maintained in the experiment. The culture was maintained at 28 °C and 180 r/min. Triplicates were applicated for each treatment.

### 3.4. Optimization of Nitrogen Source

Two different nitrogenous compounds (urea and a mixture of KNO_3_ and NaNO_3_) were tested to determine the optimal source and initial concentration of nitrogen. In the mixture of KNO_3_ and NaNO_3_, the ratio of Na^+^/K^+^ was adjusted to 6.5, which is considered the average relative atomic number ratio of Na: K in microalgae cells [28]. The urea concentration gradient (0.812, 1.218, and 1.624 g/L) and the mixed-nitrogen-source concentration gradient (1.720 g/L KNO_3_ + 0.854 g/L NaNO_3_, 2.58 g/L KNO_3_ + 1.281 g/L NaNO_3_, 3.44 g/L KNO_3_ + 1.708 g/L NaNO_3_) were applied to investigate their effects on *G. emersonii* WBG-1 growth and protein content. The culture was maintained at 28 °C and 180 r/min. Triplicates were applicated for each treatment.

### 3.5. Optimization of Fermentation Temperature

To determine the optimal temperature, a temperature gradient (25, 28, 31, 33, 35, and 37 °C) was set to investigate temperature effects on *G. emersonii* WBG-1 growth and protein content under conditions of optimal carbon and nitrogen sources at 180 r/min. Triplicates were applicated for each treatment.

### 3.6. Analytical Procedures

Cell growth was estimated by measuring the changes in dry biomass weight of the culture broth. Briefly, 10 mL of the cell cultures broth was sampled, filtered on a pre-dried GF/C filter, and washed with distilled water. Then, the filters containing algal cells were dried in an oven at 105 °C for 5 h, and the weight was measured to calculate the biomass dry weight (DW, g/L).

Algal cells were collected via centrifugation at 3000× *g* rpm for 3 min and lyophilized (−56 °C cryotrapping, 10–14 Pa vacuum) for biochemical analysis.

The glucose concentration in the medium was analyzed using a Safe-Accu UG Blood Glucose Monitoring System (Model BGMS-1; Sinocare Inc., Changsha, China) [38]. Before determination of glucose concentrations, the medium was properly diluted to make the glucose concentration within the instrument’s measurement range (0–27.8 mM). To verify the accuracy of the Blood Glucose Monitoring System, a Glucose concentration gradient was prepared using analytical glucose in the medium that was used to culture the alga, then the glucose concentrations of the medium were measured with the Blood Glucose Monitoring System, the results showed that the measured values were very close to the actual values (Full details are shown in Supplementary Table S1). The accuracy of the Blood Glucose Monitoring System was proved.

The total N concentration in the medium and dry biomass were determined by TOC cube analyzer (Elementar, Langenselbold, Germany). Lastly, the protein content of *G. emersonii* WBG-1 was calculated by multiplying the percentage of nitrogen by 6.25 [46]. In our preliminary experiment, the N distribution in *G. emersonii* WBG-1 cells was determined. It was revealed that more than 92% of the nitrogen in *G. emersonii* WBG-1 cells was in the form of protein; the proportion of non-protein nitrogen was very small (full details are shown in Supplementary Table S2). These data demonstrated that the evaluation of protein content of *G. emersonii* WBG-1 biomass via the quantification of elemental nitrogen is effective.

### 3.7. Statistical Analysis

All the treatments and control were measured in triplicate. The values were presented as the mean (n = 3) ± standard deviation. The results were analyzed using a one-way analysis of variance (ANOVA) using SPSS (version 22.0) statistical software to determine significant treatment differences (*p* < 0.05), and multiple comparison tests were performed.

## 4. Conclusions

The optimum conditions for protein production in heterotrophic cultivation of *G. emersonii* WBG-1 are as follows: glucose as a carbon source at an initial concentration of 10 g/L, urea as a nitrogen source at an initial N concentration of 54 mM, and a culture temperature of 35 °C. The maximum biomass and protein content reached 4.85 g/L and 66.14%. In addition, the highest protein yields and productivity reached 3.15 g/L and 1.52 g/L/d in batch cultures under optimal conditions, respectively. The protein content obtained under this set of conditions was significantly higher than that reported for *Chlorella* heterotrophic cultures and much higher than that reported for specially established technologies to increase the protein content, such as two-stage heterotrophic, sequential heterotrophic-autotrophic, heterotrophy–dilution–photoinduction, and mixotrophic processes, and this result lays the foundation for improving protein yield and productivity. Therefore, we conclude that *G. emersonii* WBG-1 is a promising candidate for microalgal protein production via heterotrophic culture and has great potential for application in protein mass production.

## Figures and Tables

**Table 1:** Effects of three carbon sources on the biomass and protein production of *Graesiella emersonii* WBG-1.

Carbon Source	Initial Concentration (g·L^−1^)	Biomass (g·L^−1^)	Protein Content (%, DW)	Protein Yield (g·L^−1^)
Glucose	30	15.36 ± 0.23	34.19 ± 0.53	5.25 ± 0.12
Sodium acetate	30	4.45 ± 0.15	38.89 ± 0.42	1.73 ± 0.06
Sucrose	30	—	—	—

Note: “—” indicating no data available owing to failed grow of alga.

**Table 2:** Comparison of protein content of microalgae cultured under different technical conditions.

Species	Cultivation Mode	Protein Content (% DW)	Reference
*Graesiella emersonii* WBG-1	heterotrophic	66.1	This study
*Chlorella* sp. CMBB276	heterotrophic	37.3	[38]
*Chlorella vulgaris*	heterotrophic	48.7	[43]
*Chlorella sorokiniana*	heterotrophic	16.3	[16]
*Chlorella vulgaris*	heterotrophic	36.5	[44]
*Chlorella vulgaris*	two-stage heterotrophic	44.3	[21]
*Chlorella vulgaris*	two-stage heterotrophic	59.8	[37]
*Chlorella*	sequential heterotrophic/autotrophic	60.1	[18]
*Chlorella vulgaris*	Heterotrophy–dilution–photoinduction	50.9	[20]
*Chlorella vulgaris*	mixotrophic	58.4	[19]

## Data Availability

Data are available from the authors upon reasonable request.

## Supplementary Materials

The following supporting information can be downloaded at: https://www.mdpi.com/article/10.3390/plants12122255/s1, Table S1: The protein nitrogen and non-protein nitrogen in *Graesiella emersonii* WBG-1 biomass; Table S2: Comparison of measured concentrations to actual concentrations of glucose.
