# Supplementary material for: Optimization of Heterotrophic Culture Conditions for the Algae Graesiella emersonii WBG-1 to Produce Proteins
Source: Plants (Basel). 2023 Jun 9;12(12):2255. doi: 10.3390/plants12122255 (PMC10303403; doi:10.3390/plants12122255)
Supplement: Supplementary file 1 [file plants-12-02255-s001.zip › plants-2375623-supplementary.pdf]

Table S1. The protein nitrogen and non-protein nitrogen in *Graesiella emersonii* WBG-1 biomass.

	**N content （mg g^{-1}）**	**N content (%)**
TN	87.79	100
N in Protein	80.86	92.11
N in Chl *a* and Chl *b*	1.84	2.10
DIN （Dissolved Inorganic Nitrogen）	0.0022	0.0025
N in Nucleic acids	5.09	5.80

Table S2. Comparison of measured concentrations to actual concentrations of glucose

**Actual concentration (g/L)<br>(mM)**	**10<br>55.55**	**5<br>27.75**	**2.5<br>13.88**	**1<br>5.55**
Measured concentration (mM)	55.13±0.50	27.40±0.50	13.80±0.26	5.64±0.10
